# Supplementary material for: Exploring the Interplay of RUNX2 and CXCR4 in Melanoma Progression
Source: Cells. 2024 Feb 27;13(5):408. doi: 10.3390/cells13050408 (PMC10930675; doi:10.3390/cells13050408)
Supplement: Supplementary file 1 [file cells-13-00408-s001.zip › Supplemental Figure S2.pdf]

To correctly identify the bands, we used the following protein markers. These markers are not well visualized in chemiluminescence. Therefore, we mark the membrane at the right positions to cut it accurately and/or identify the protein highlighted in chemiluminescence.

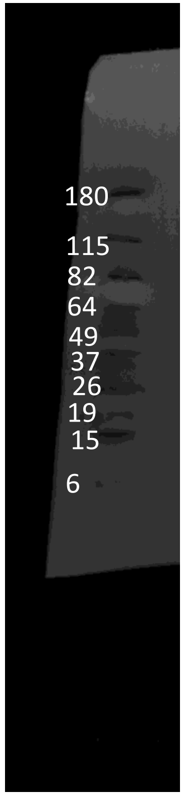

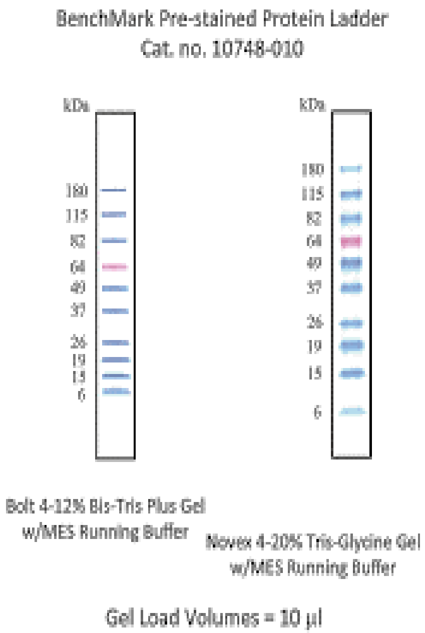

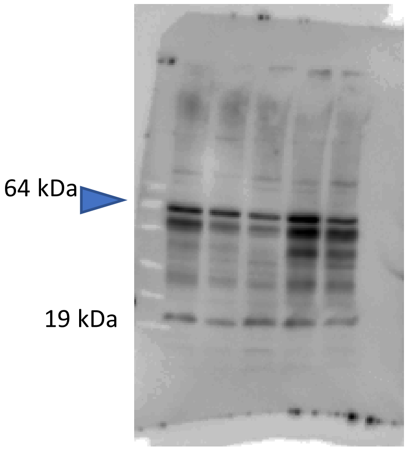

Figure 1D RUNX2 lane 1, 2, 3, 4, 5

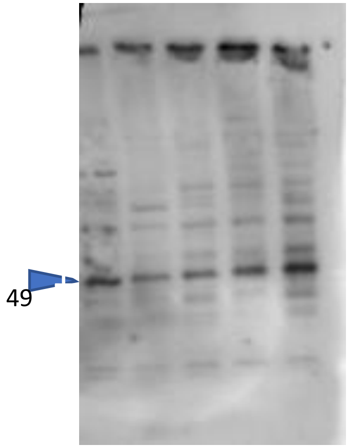

Figure 1D MMP3 lane 2,3,4,5,

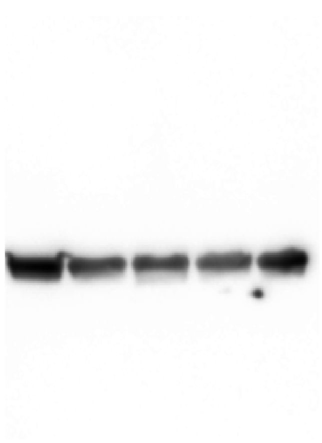

Figure 1D B actin lane 1,2,3,4,5

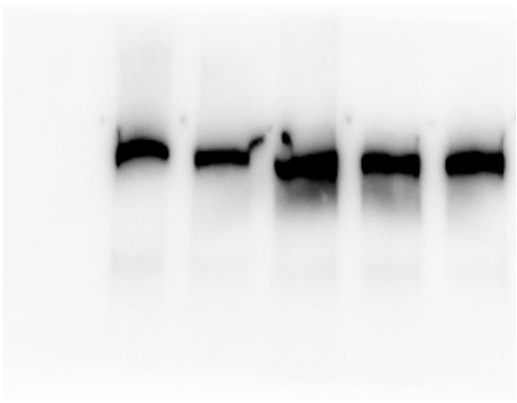

Figure 1E CXCR4 lane 1, 2,3,4,5

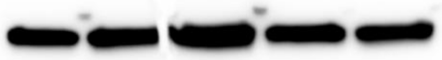

Figure 1E B actin lane 1, 2,3,4,5

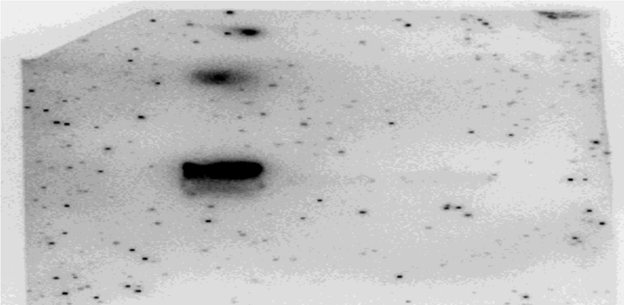

Figure 2A RUNX2 lane 1,2,3,

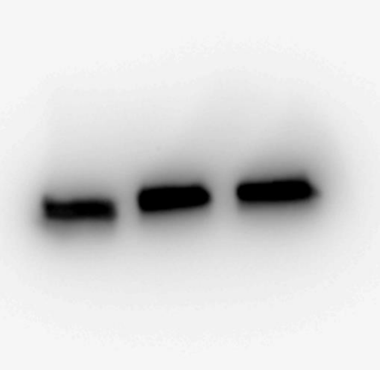

Figure 2A B Actin lane 1,2,3

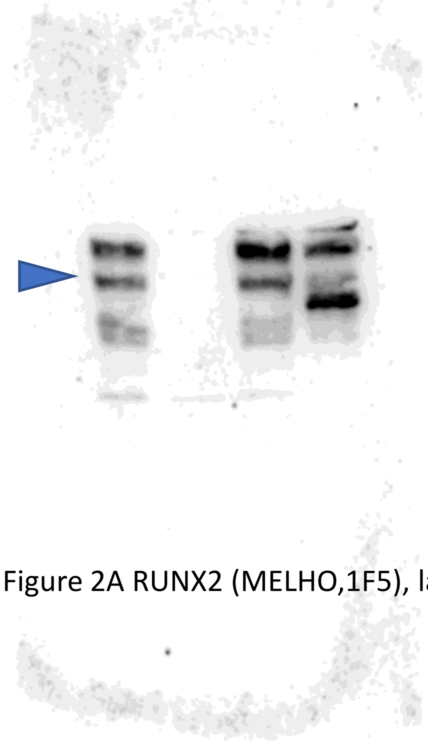

Figure 2A RUNX2 (MELHO,1F5), lane 1,2

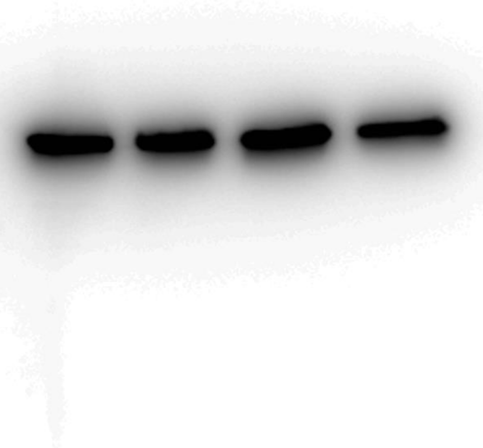

Figure 2A B actin (MELHO,1F5), lane 1,2

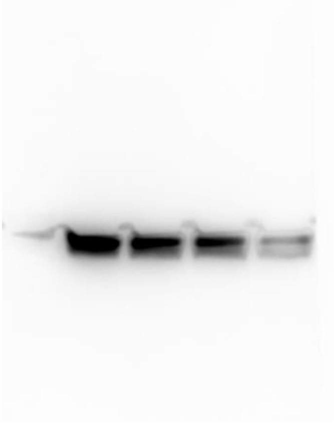

Figure 2C CXCR4 lane 2,3,4,5

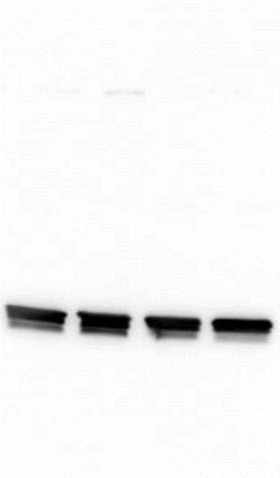

Figure 2C B actin lane 1,2,3,4

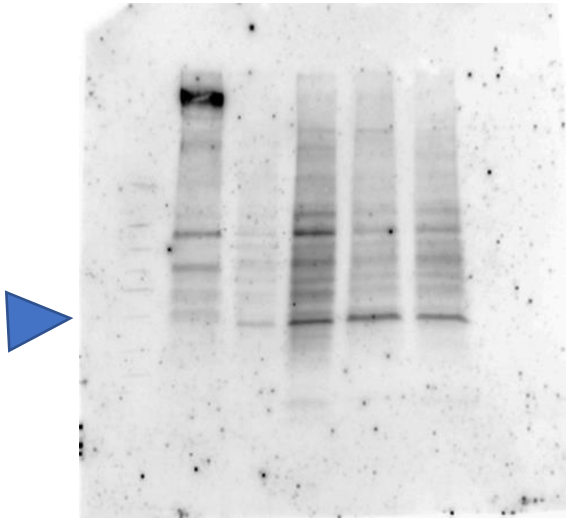

Figure 3B RANKL lane 2,3,4,5

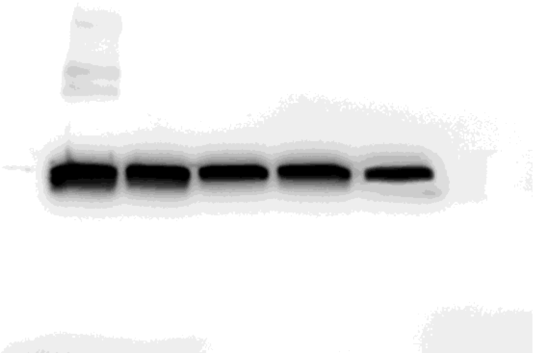

Figure 3B B actin lane 2,3,4,5

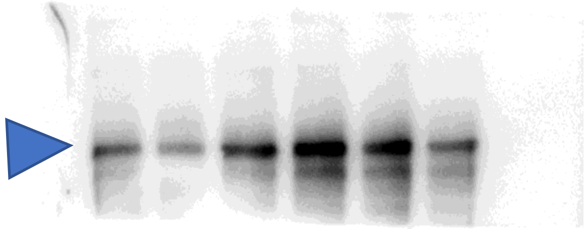

Figura 3C CXCR4 Lane 2, 3, 4

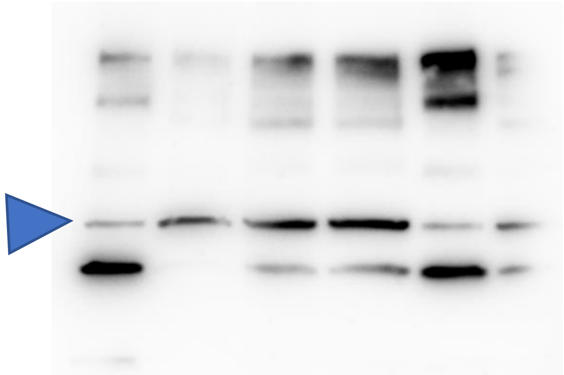

Figura 3C MMP3 Lane 2, 3, 4

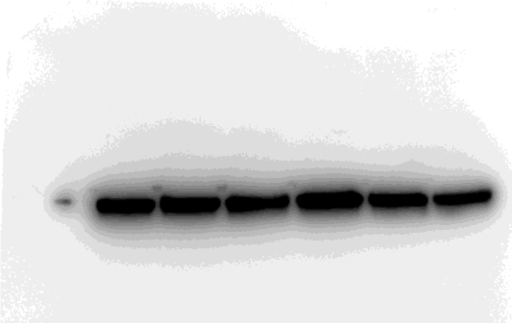

Figure 3CB actin lane 2,3,4

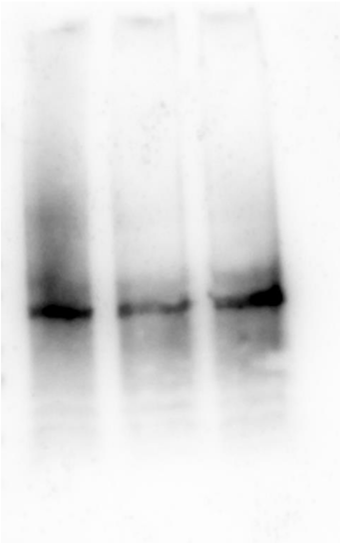

Figure 4d CXCR4 lane 1,2,3

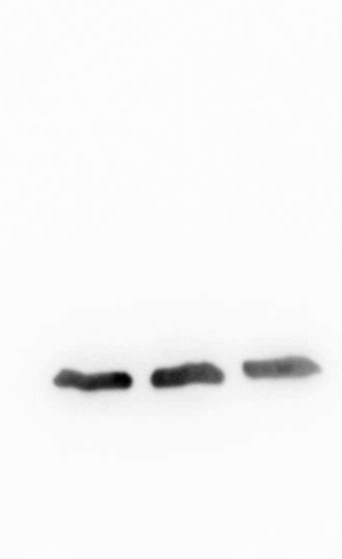

Figure 4d B actin lane 1,2,3

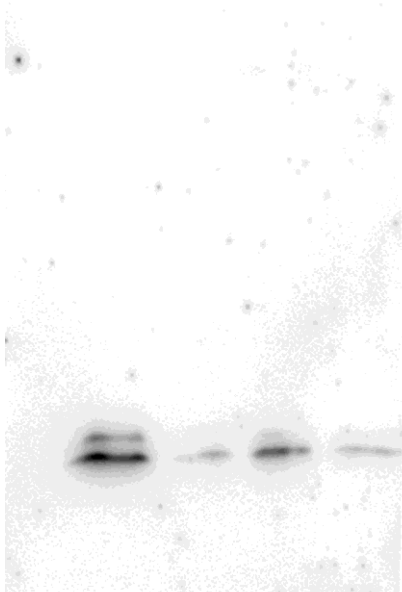

Figure 5A LC3 lane 2,3,4,5

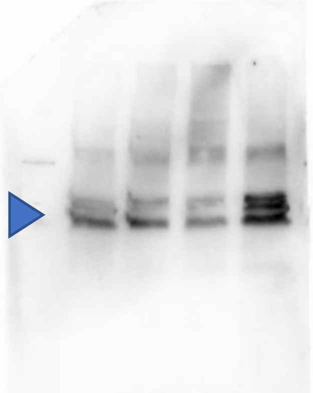

Figure 5A P62 lane 2,3,4,5

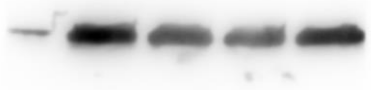

Figure 5A B actin lane 2,3,4,5

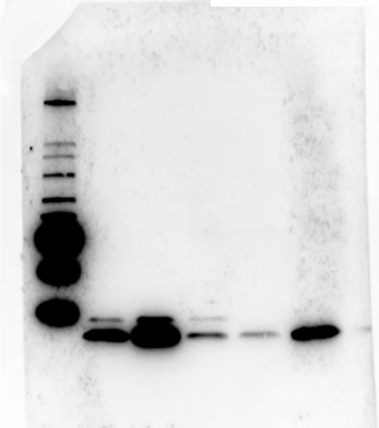

Figura 5B LCB Lane 2-7

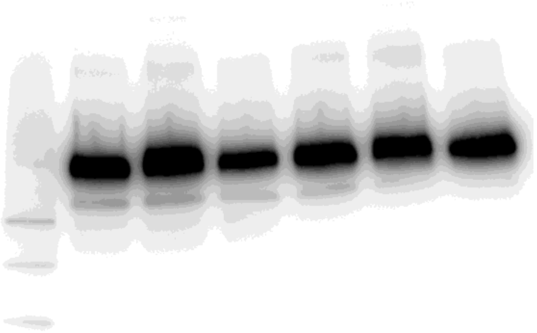

Figura 5B P62 lane 2-7)

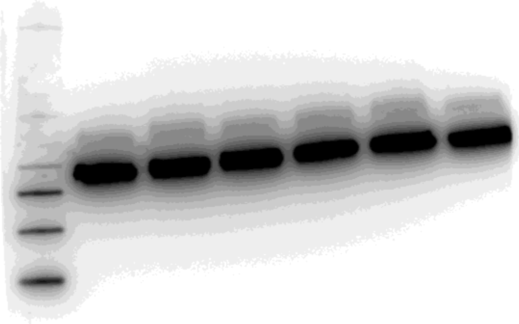

Figura 5B actina (lane 2-7)

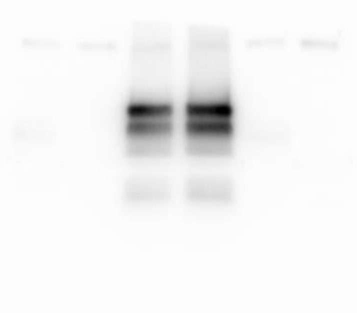

Figura 5C RUNX2 lane 2,3,4

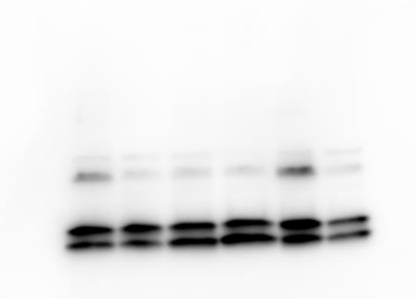

Figura 5C LC3 lane 2,3,4

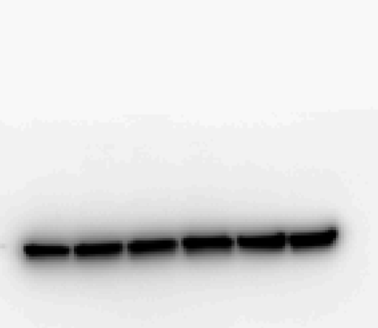

Figura 5C B actin lane 2,3,4

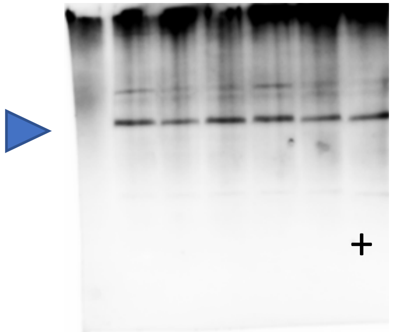

Figura 5D Beclin lane 1,2,3,4

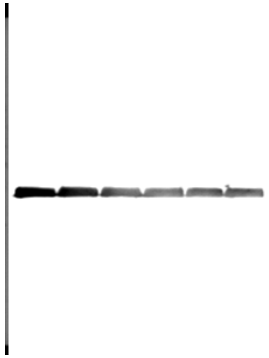

Figura 5D Bactin lane 1,2,3,4

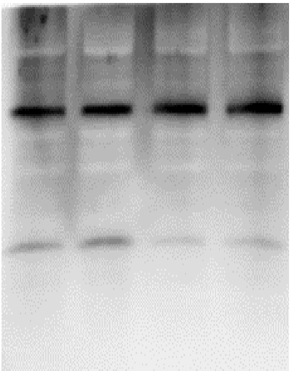

Figure 6A PS6K lane 1,2

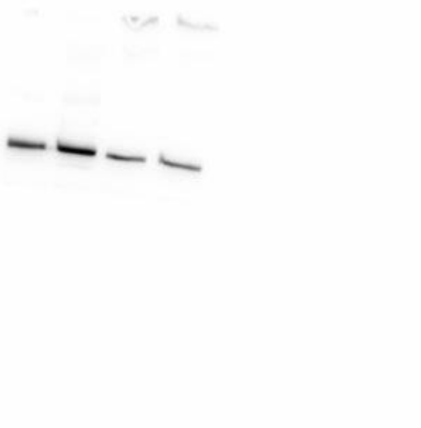

Figure 6A P-PS6K lane 1,2

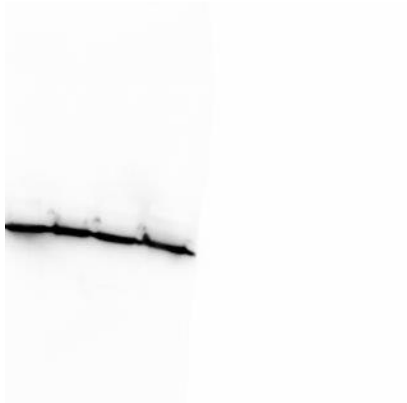

Figure 6A B actin lane 1,2

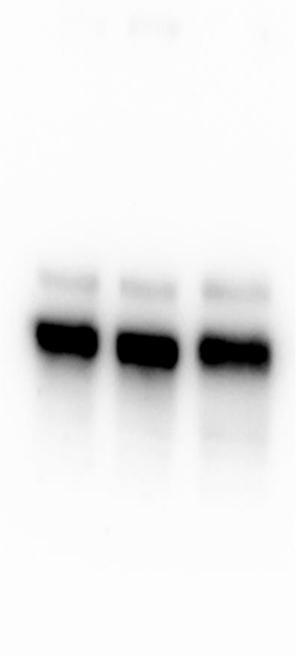

Figure 6B mTOR lane 1,2

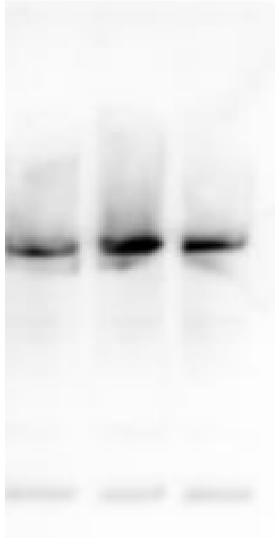

Figure 6B P-mTOR, lane 1,2

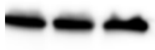

Figure 6B B actin lane 1,2

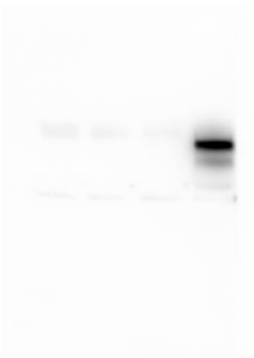

Figure 7A RUNX2 lane 3 and 4

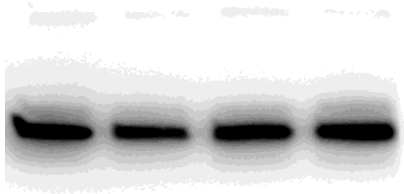

Figure 7A p70SK lane 3 and 4

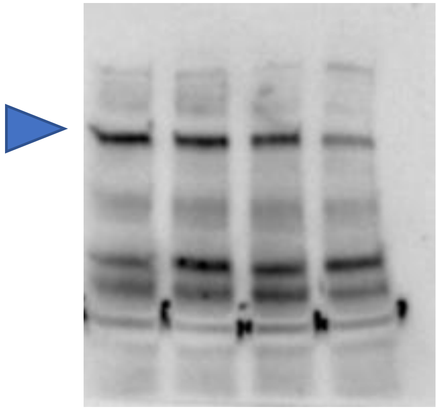

Figure 7A P-p70SK lane 3 and 4

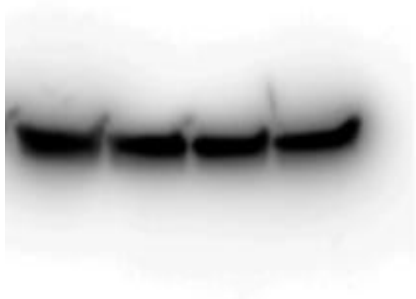

Figure 7A B Actin lane 3 and 4

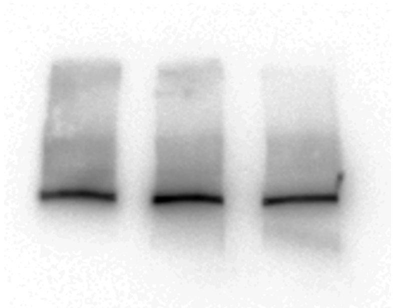

Figura 7B mTOR (lane 1, 2, 3)

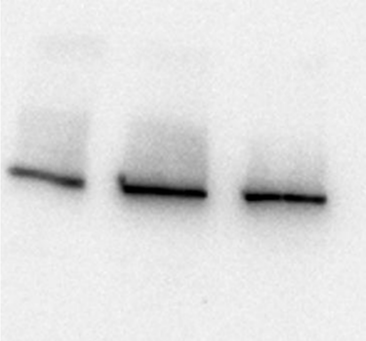

Figura 7B P-mTOR (lane 1, 2, 3)

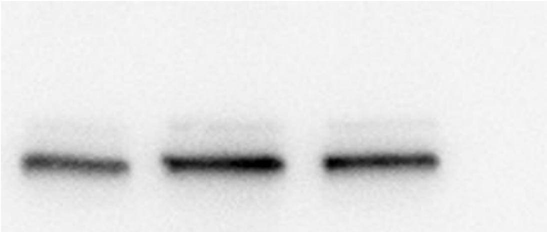

Figura 7B PS6K (lane 1, 2, 3)

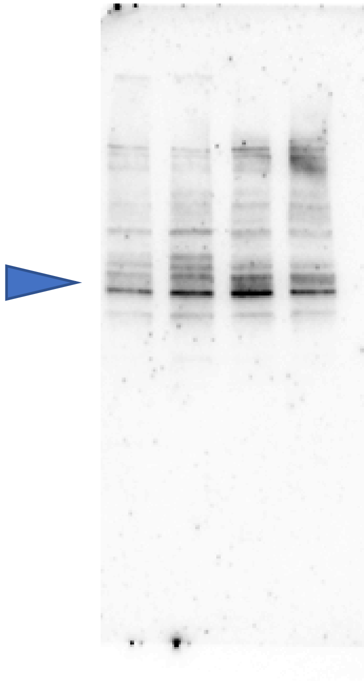

Figura 7B P-PS6K (lane 1, 2, 3)

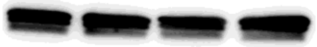

Figura 7B B actin (lane 1, 2, 3)
