# Supplementary figures and images for: Exploring the Interplay of RUNX2 and CXCR4 in Melanoma Progression
Source: Cells. 2024 Feb 27;13(5):408. doi: 10.3390/cells13050408 (PMC10930675; doi:10.3390/cells13050408)

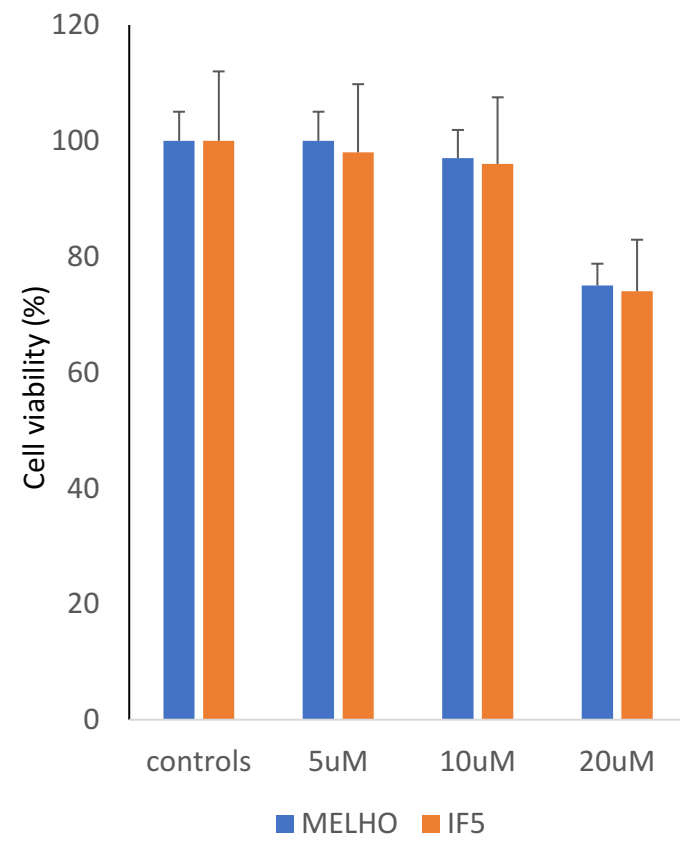

Supplement: Supplementary file 1 [file cells-13-00408-s001.zip › Supplemental Figure S1.pdf]
